# Management options for low-dose methotrexate-induced oral ulcers: A systematic review

**DOI:** 10.4317/medoral.22851

**Published:** 2019-03

**Authors:** Cintia Chamorro-Petronacci, Abel García-García, Alejandro-Ismael Lorenzo-Pouso, Francisco J. Gómez-García, María-Elena Padín-Iruegas, Pilar Gándara-Vila, Andrés Blanco-Carrión, Mario Pérez-Sayáns

**Affiliations:** 1 PhD, DDS, Oral Medicine, Oral Surgery and Implantology Unit, Faculty of Medicine and Dentistry, Institute for Health Research of Santiago (Instituto de Investigación Sanitaria de Santiago, IDIS), Santiago de Compostela, Spain; 2 MD, PhD, Oral Medicine, Oral Surgery and Implantology Unit, Faculty of Medicine and Dentistry, Institute for Health Research of Santiago (Instituto de Investigación Sanitaria de Santiago, IDIS), Santiago de Compostela, Spain; 3 DDS, MSc, Oral Medicine, Oral Surgery and Implantology Unit, Faculty of Medicine and Dentistry, Institute for Health Research of Santiago (Instituto de Investigación Sanitaria de Santiago, IDIS), Santiago de Compostela, Spain; 4 DDS, PhD, Oral Medicine, The Murcia Institute of Biomedical Research (Instituto Murciano de Investigación Biomédica, IMIB), Murcia, Spain; 5 MD, PhD, Human Anatomy and Embryology area, Faculty of Physiotherapy, Department of Functional Biology and Health Sciences, Pontevedra, Vigo University, Spain; 6 DDS, PhD, Oral Medicine, Oral Surgery and Implantology Unit, Faculty of Medicine and Dentistry, University of Santiago de Compostela, Santiago de Compostela, Spain; 7 MD, PhD, Oral Medicine, Oral Surgery and Implantology Unit, Faculty of Medicine and Dentistry

## Abstract

**Background:**

Oral ulcers caused by methotrexate (MTX) at low doses are a known side effect of this drug. Although increasingly more patients are medicated with MTX, these painful ulcers, without traumatic origin and resistant to any type of treatment, are not usually identified by health professionals as a side effect of the medication.

**Material and Methods:**

In the absence of a consensus protocol for the effective treatment of oral lesions produced by MTX, the objective of this article was to review and analyse the information from articles related to oral ulcers produced by low-dose MTX and to record the clinical management performed and the MTX dose given to the patient. Data sources - Medline, Web of Science, and Cochrane Library. Participants - Patients treated with low-dose MTX (less than 25 mg/week). Interventions - Management of oral lesions caused by MTX. Study eligibility criterion, study appraisal and synthesis method: An initial search was carried out in the aforementioned databases with the terms ‘methotrexate AND oral OR ulcer’. The search was carried out using both medical subject heading (MeSH) terms and a free search between January 2003 and January 2018. Of the results obtained, two independent researchers analysed abstracts that met the search criteria, that is, those that mentioned oral ulcers produced by MTX at low doses. Next, both researchers read the complete article and determined whether it met the following inclusion criteria: written in English, specified the dose of MTX prescribed for the patient and specified the protocol of action for the ulcers. A third investigator acted as a mediator in cases of dispute. Agreement was calculated using Cohen’s kappa coefficient, with a k value of 0.82. The Preferred Reporting Items for Systematic Reviews and Meta-Analyses (PRISMA) guide for systematic reviews was used.

**Results:**

The initial search resulted in a total of 66 articles, of which 30 were selected to assess their inclusion in this study. Finally, 16 met the inclusion criteria. Using the Pierson and Newcastle-Ottawa scales and Bradford Hill criteria modified for studies of case series and “in relation to a case”, 2 were rated as high quality, 2 were rated as low quality and 12 were rated as medium quality. The limitations of this study are based on the fact that all of the articles available to carry out the systematic review were “in relation to a case or series of cases”, with the heterogeneity of data that this implies.

**Conclusions:**

Evidence on the management of oral ulcers in the oral cavity produced by MTX at low doses is scarce due to the heterogeneity of data and the measures adopted in the selected studies. Therefore, it seems that this management is relegated to the perception of the clinician rather than to a specific protocol of action. Studies with a longer follow-up duration and larger sample size are needed to guide different health professionals on the management of these lesions.

** Key words:**Methotrexate, Oral ulcers, Low-dose methotrexate.

## Introduction

Methotrexate (MTX) is an immunomodulator that functions as an antagonist of folic acid and is widely used at high doses as a chemotherapeutic agent for the treatment of lymphomas, leukaemia and some solid tumours. MTX binds irreversibly to dihydrofolate reductase by exerting antiproliferative activity (1). At low doses (no more than 25 mg/week), its main effect is anti-inflammatory and is used for the treatment of several autoimmune diseases, such as rheumatoid arthritis, systemic lupus erythematosus, pemphigoid, polymyositis or psoriasis (2). Although its use is recommended in different guidelines for the treatment of diseases previously mentioned for more than 50 years and is considered safe at low doses, data on the efficacy and safety of MTX are scarce (3,4).

In blood, MTX is conjugated with albumin and excreted via the kidneys. Interaction with drugs that decrease the renal elimination of MTX, that inhibit the synthesis of folic acid or that decrease the binding of MTX to proteins can trigger adverse effects (5). Concomitant factors, such as old age, other drugs or kidney failure, can increase the toxicity of the drug (6).

This drug usually affects tissues of rapid tissue renewal, such as the bone marrow or the gastrointestinal tract. Oral ulcers may appear in 11%-17% of patients treated with MTX at low doses (2,3,7). The most common effects of MTX at low and high doses are acute myeloid suppression, gastrointestinal disorders, hepatotoxicity and acute kidney failure (4). Other common side effects in patients treated with low-dose MTX include nausea, diarrhoea and abdominal pain (2) and the recorded treatment abandonment rate is between 3%-6% (3,8).

Adverse reactions from long-term low-dose MTX treatment may be present in 30% to 80% of patients, and acute toxicity in patients taking MTX at low doses may, in some cases, be life-threatening (9).

The most recent publications classify the adverse effects of MTX into four categories: category A dose-dependent, category B or idiosyncratic, category C or effects due to cumulative dose, and category D or delayed effects after cessation of treatment. Category A includes mucocutaneous and gastrointestinal toxicity and occasionally pancytopenia, which may also be due to idiosyncratic effects (category B). The cumulative dose includes chronic liver and lung toxicity, although the association between liver toxicity and cumulative dose of MTX is controversial. Delayed effects (category D) include teratogenicity (6).

For the treatment of acute toxicity due to MTX, several measures are proposed. Yélamo et al. explained that folic acid supplementation improves gastrointestinal tolerance and prevents haematological toxicity (6). These authors also recommend that in the presence of pancytopenia, mucositis or cutaneous ulcers, MTX should be eliminated since its effects can be life-threatening, with hydration causing an increase in the renal elimination of the drug and the addition of folic acid as an antidote being important. It also specifies that once the toxicity has passed, MTX can be reintroduced.

Other authors such as Kalantzis *et al.* summarized a series of measures for the treatment of acute MTX toxicity, including cessation of treatment, reduction of its dose, folic acid complement or a combination of measures. However, the authors did not explain under what circumstances each measure must be applied or what benefits they provide with respect to other measures (8).

Nevertheless, MTX at low doses is the first line of action for the treatment of different autoimmune pathologies in European guidelines (10). The prevalence of psoriasis in the Spanish population is 2.4% (11) and some authors believe that the adverse effects of MTX are being underestimated by dermatologists, pathologists, dentists or maxillofacial specialists (7).

In the absence of a consensual action protocol for the effective treatment of oral lesions caused by MTX at low doses, the objective of this article was to review and analyse the information from articles related to patients who have suffered oral ulcers produced by low-dose MTX, recording the method of action carried out and the MTX dose given to the patient. The PECOS mnemonic was used to formulate the following question: In patients with oral ulcers who take MTX at low doses, what is the most effective management for their complete cure? To address this question, we carried out a systematic review on oral ulcers produced by MTX at low doses.

## Material and Methods

-Protocol and registration:

The design of this study was carried out by CHP and is registered in PROSPERO (CRD42018103692). The review was carried out following PRISMA guidelines and the PECOS method (12): patients with oral ulcers taking low-dose MTX (P=patient); withdrawing MTX (E=exposure); non-ceasing MTX (C=comparison); time healing ulcers (O=outcome); case report (S= type of study).

-Eligibility criteria, information sources and search.

Exclusion criteria were as follows: articles that did not address ulcers produced by MTX; abstract not available; written in a language other than English or Spanish; prescribed dose for the MTX patient was unspecified; absence of a protocol to follow for healing oral ulcers; and sex or age of the patient was unspecified.

We carried out a literature search in PubMed, Embase and Web of Science to identify relevant studies on ulcers produced by MTX written in English between January 2003 and January 2018 using the terms ‘methotrexate AND oral OR ulcer’. Medical subject heading (MeSH) terms and free search were also used. All articles on “in relation to a case”, cohort studies and case-control studies were included.

-Study selection:

From the results obtained from the search, two independent researchers (MPS and CHP) analysed abstracts that met the search criteria, that is, abstracts that mentioned oral ulcers produced by MTX at low doses. Then, both researchers read the complete article and determined whether it met the following inclusion criteria: written in English, specified the dose of MTX taken by the patient and specified the protocol of action for the ulcers. A third investigator (AGG) acted as a mediator in cases of dispute. Agreement was calculated using Cohen’s kappa coefficient, with a k value of 0.82.

-Data collection process:

Data retrieved from all articles were collected by both researchers (in duplicate) independently and corroborated by the third party, who acted as a mediator in cases of discrepancy or lacked agreement.

-Data items:

The following information was extracted from each study: first author, year of publication, sex and age of the patient, type of lesion (single or multiple), location, possible cause, time of evolution, management of the lesion, restoration of MTX without relapse of the lesion, disease for which MTX is taken, HSV (herpes simplex virus), EBV (Epstein-Barr virus), CMV (cytomegalovirus), folic acid intake prior to the onset of the lesion, histological analysis, MTX dose, oral or subcutaneous, length of time taking MTX (months), time to healing, kidney involvement, liver involvement, myelosuppression, smoker and cigarettes/day, other medications, and follow-up time.

If the time taking MTX was less than one month, it was considered 0, indicating that the appearance of ulcers was due to the recent introduction of MTX as a treatment.

-Risk of bias in individual studies:

The methodological quality of the included studies and the possibility of bias were assessed using the modified Newcastle-Ottawa, Pierson and Bradford Hill scales for case series and “in relation to a case” studies (13). The authors of this scale recommend assessing the quality of the studies according to four categories, selection, ascertainment, causality and reporting, with eight specific questions to answer giving low (1-3 questions), medium (4-6) questions) and high (7-8 questions) quality values. This analysis was carried out independently by each of the two investigators and in cases of disagreement, the third acted as a mediator.

-Summary measures:

All variables were collected in a database and analysed with SPSS v. 20.0 (IBM Corp. Released 2011. IBM SPSS Statistics for Windows, Version 20.0. Armonk, NY: IBM Corp.). For the univariate description, we used basic descriptive statistics, such as mean, standard deviation, frequency and percentage.

## Results

**Figure 1:**
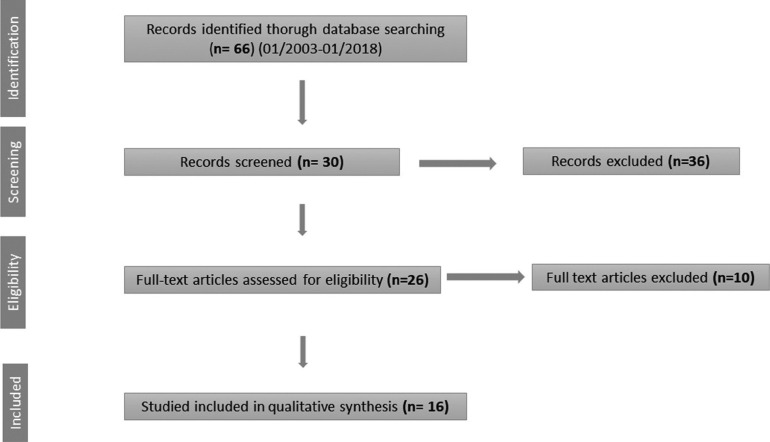
Flow Diagram.

-Study selection:

The search process involved a total of 66 articles, of which 36 were excluded because they were not articles on oral ulcers produced by MTX. After reading the latter, 16 met the aforementioned inclusion criteria (Fig. 1).

-Study characteristics:

Of the 16 articles, 2 were rated as high quality (12.5%), 2 as low quality (12.5%) and 12 as medium quality (75%) ( Table 1). The summary of data obtained from the patients extracted from the studies can be seen in  Table 2.

**Table 1:**
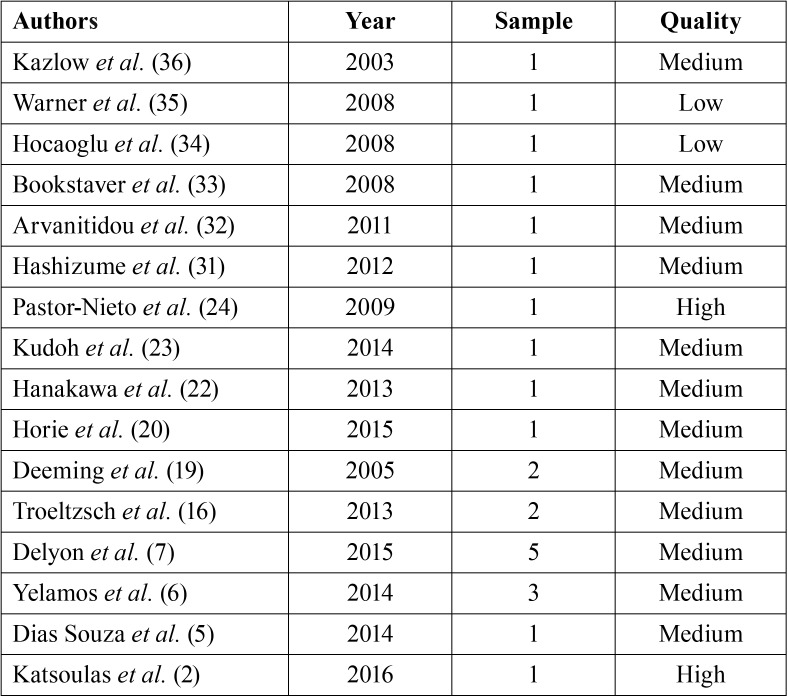
Quality of the articles included in this systemic review.

**Table 2:**
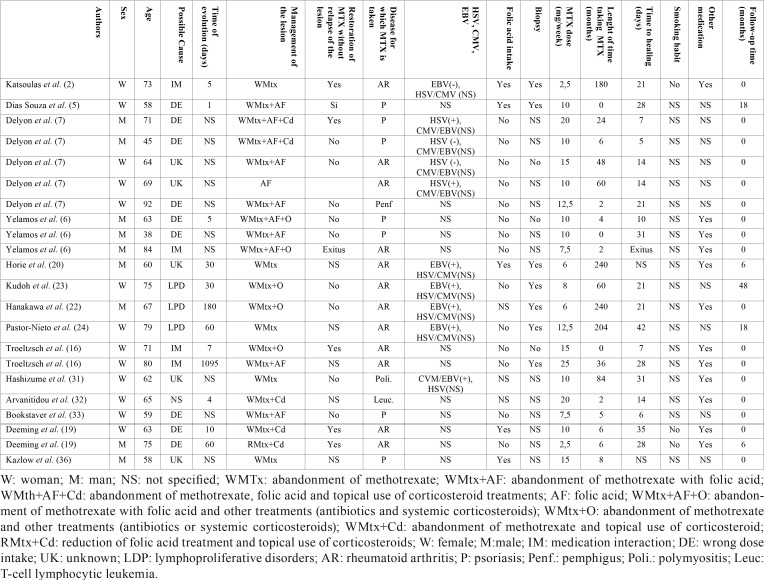
Data patients extracted from the studies included in this systemic review.

The 16 articles involved a total of 24 patients and the epidemiological data are summarized in  Table 3.

**Table 3:**
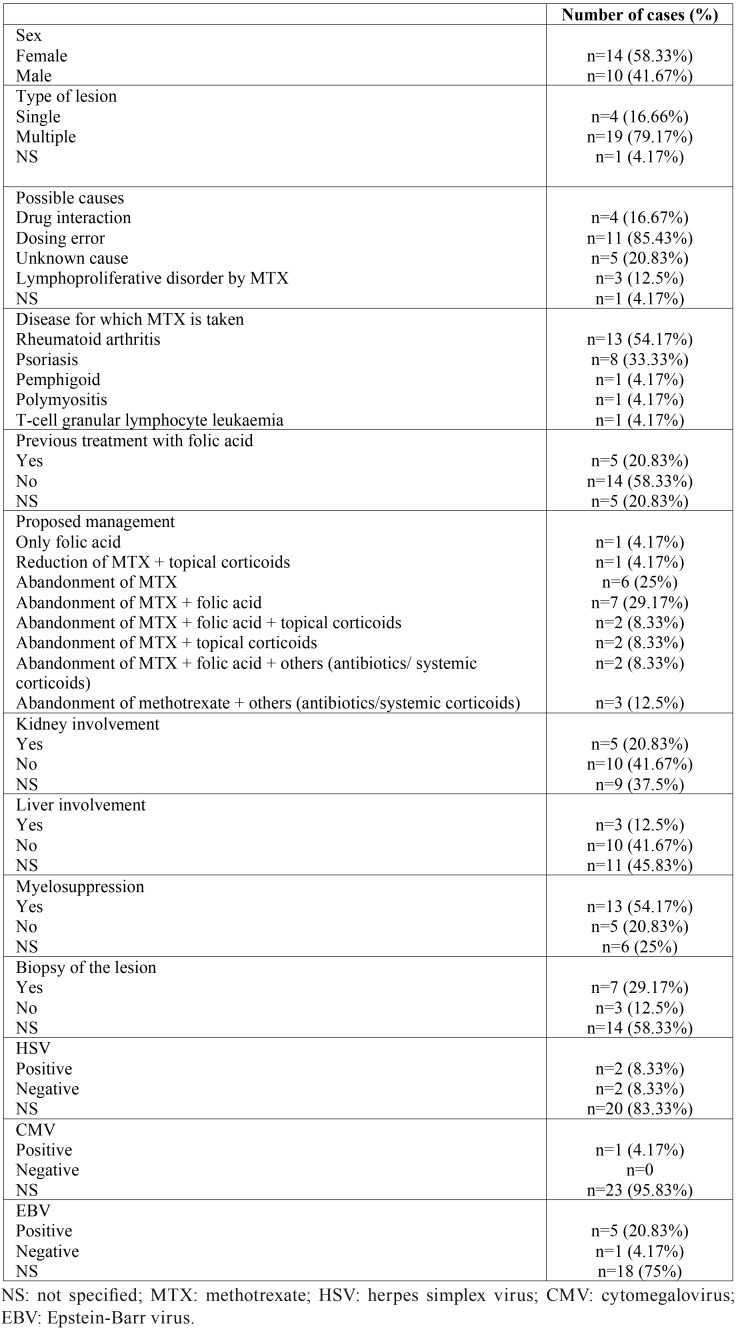
Descriptive statistics.

The mean age of the patients affected by oral ulcers was 65.45 years (standard deviation [SD]=13.20), with a range of 36-92 years.

In 8 patients, the location of the lesion was not specified (33.33%). In the four patients with a single lesion, two appeared in the retromolar area, another in the keratinized gingiva and another in the lip.

All patients except for one took MTX subcutaneously (4.17%) and the rest took MTX orally (95.83%).

The mean time of evolution of the lesion before being treated by the medical service in patients who specified the time (50%) was 123.91 days (SD=309.81). However, one patient lived with the lesions for three years without being treated, which distorts the results. If we exclude this patient, the mean time of evolution of the lesions was 35.63 days (SD=52.57).

The mean time of the patients in which the months were specified taking MTX (23/24) was 52.91 months (SD=80.75), with a range of 0-240 months.

Only 3 of the 24 patients specified their smoking habit and were non-smokers (12.5%). Only 12 of the 24 patients specified any concomitant medication (50%).

Regarding the healing time of oral ulcers in days, in 3 patients the healing time was not specified and one patient died due to complications of MTX poisoning. In the remaining 20 patients, the mean healing time was 19.9 days (SD=10.63).

The average dose of MTX prescribed to each patient was 10.93 mg/week (SD=5.45).

**Figure 2:**
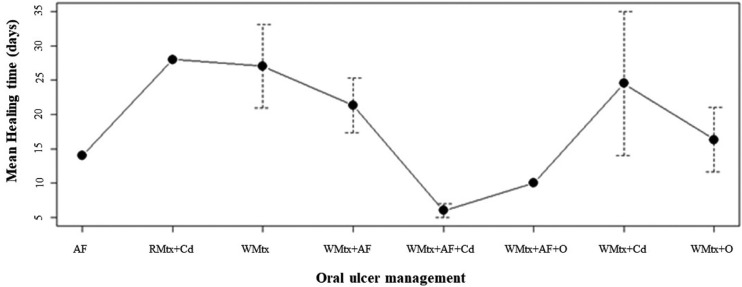
Plot of means of oral ulcer management. WMTx: abandonment of methotrexate; WMtx+AF: abandonment of methotrexate with folic acid; WMth+AF+Cd: abandonment of methotrexate, folic acid and topical use of corticosteroid treatments; AF: folic acid; WMtx+AF+O: abandonment of methotrexate with folic acid and other treatments (antibiotics and systemic corticosteroids); WMtx+O: abandonment of methotrexate and other treatments (antibiotics or systemic corticosteroids); WMtx+Cd: abandonment of methotrexate and topical use of corticosteroid; RMtx+Cd: reduction of folic acid treatment and topical use of corticosteroids.

The most common management for healing oral ulcers was the abandonment of MTX and treatment with folic acid (29.17%), followed by simply the abandonment of MTX (25%). MTX was withdrawn in 12.5% of cases and patients were given other treatments, such as antibiotics and systemic corticosteroids. Other authors combined the cessation of MTX with folic acid and oral corticosteroid treatments (8.33%) or the cessation of MTX with folic acid and other drugs, such as systemic corticosteroids and antibiotics (8.33%), or simply the cessation of MTX combined with oral corticosteroids (8.33%). The least frequently used measures were simple treatment with folic acid (4.17%) and the reduction of folic acid treatment and the use of corticosteroids (4.17%). The average time of total healing of the oral lesions in each proposed management can be seen in Figure 2.

In total, 50% of the studies analysed specified the concomitant medication that the patient was taking (apart from MTX).

The number of patients who returned to MTX without suffering the reappearance of oral ulcers was 6 (25%), while 8 patients did not specify their return (33.33%) and 10 did not resume MTX (41.67%).

-Risk bias across studies:

All studies that fulfilled the inclusion criteria were “in relation to a case” and most included patients who were referred or admitted urgently to a maxillofacial or emergency service in the hospital setting. Therefore, data on the patient’s history depended, to a large extent, on data that he/she contributed and not to previous monitoring. The follow-up duration was longer than one month in only 5 (20.83%) patients and the mean follow-up time of these patients was 19.2 months (SD=17.81).

## Discussion

The search to identify oral ulcers managed by MTX yielded a total of 16 articles, where most were classified as medium quality, with a total of 24 patients.

Despite the heterogeneity of the proposed management in the different studies, the majority (29.17%) opted for the abandonment of MTX and additional treatment with folic acid, followed by the simple abandonment of MTX (25%).

Studies on the adverse effects of MTX at low doses conclude that there is usually a dose-dependent relationship (category A), but there are insufficient data to differentiate MTX stomatitis from other entities, such as lichenoid reactions (8).

Authors such as Avery *et al.* claim that the most frequent reasons for MTX toxicity are an increase in exposure to the drug due to an error in the dose administered, to its interaction with other medications (especially antibiotics and nonsteroidal anti-inflammatory drugs, NSAIDs), renal failure, infections or advanced age (14,15). This observation coincides with our results because the majority of oral ulcers due to MTX were associated with erroneous intake of the dose (85.43%) and drug interaction (specifically with ciprofloxacin, ibuprofen and omeprazole) that occurred in 16.67% of the patient cases. Taking into account the non-negligible number of drugs that can interact with MTX increasing its toxicity, one bias to be taken into account is that half of the studies included in this review did not record the patient’s medication or did not account for it when treating oral ulcers, since we did not observe the cessation of any medication that could interact with MTX as a proposed management for oral ulcers in any of the articles studied.

The number of patients who returned to MTX without suffering a reappearance of oral ulcers was 6 (25%), while 8 did not specify (33.33%) and 10 did not resume MTX (41.67%). None of the articles specified why the patient decided to retake MTX, and there is controversy among authors who suggest that it can be safely retaken (6) and those who have registered a 50% relapse of patients a few days after the first event (2).

The most frequent localizations of the oral lesions produced by MTX are the lingual dorsum, the hard palate and the gingiva (16). In those single lesions, which were observed in 4 patients (16.66%), 2 appeared in the retromolar area, one in the keratinized gingiva and another in the lip. In cases of multiple lesions, ulcers appeared in labial, oral and keratinized mucosa.

Despite the effects that MTX toxicity can have on patients, MTX is more effective than other disease-modifying antirheumatic drugs (DMARDs), with more than half of patients experiencing at least one moderate improvement (8). This means that the complete cessation of MTX, without resuming, limits the options of patients who require treatment for their autoimmune disease.

Another adverse effect of MTX is the iatrogenic association with lymphoproliferative disorders (LPDs). These disorders encompass multiple conditions in which lymphocytes are produced in excessive amounts, as in lymphomas, and are more frequent in immunocompromised patients. Since MTX is an immunosuppressive agent, it is also considered a common cause of iatrogenic LPDs associated with immunodeficiencies (17). Iatrogenic lymphoproliferative disorders are atypical lymphoid proliferations or lymphomas that affect patients treated with immunosuppressants. In 2008, the World Health Organization (WHO) classified iatrogenic immunodeficiency produced by MTX within this group (21). A higher incidence of lymphomas has been observed in patients with rheumatoid arthritis treated with MTX (22). In our review, LPDs due to MTX were associated with the appearance of oral ulcers in 3 patients (12.5%).

The clinical and histological characteristics of oral ulcers produced by MTX are not always diagnostic and an extensive clinicopathological investigation is necessary to exclude lymphoproliferative disorders; histopathological analysis has revealed a broad spectrum, ranging from non-specific ulceration, lichenoid reactions or lymphoproliferative disorders (2). Of the studies analysed in this literature review, only 7 (29.17%) performed a biopsy of the oral ulcer and the results were different: polymorphous lymphohistiocytic proliferation with atypical binuclear lymphocytes (2); area covered by exudate and regenerating epithelium, ectasia in proliferative vessels and neutrophils at the bottom of the lesion (5); regenerative basal layer with multinuclear keratinocytes (7); proliferation of large lymphoid cells with excised nuclei containing conspicuous nucleoli (17); atypical lymphocytic infiltrate with prominent nucleoli in submucosal granulation tissues (18); numerous proliferations of large atypical lymphoid cells with small lymphocytes (19); dense infiltrate composed of large lymphoid cells with irregular margins that occupy the entire thickness of the mucous membrane (20); and irregular and hyperplastic epithelial layers with apoptotic cells, atypical nuclei and a lichenoid appearance (21).

The different responses to MTX treatment are believed to be due to the heterogeneity between patients related to the polymorphisms of the genes involved in the metabolism of MTX or folic acid (22), the phenotype of the disease, and the previous duration of the disease, in addition to the factors associated with the absorption of the drug and its kinetics (4).

In 2010, Dojcinov *et al.* proposed positive mucocutaneous ulcers for EBV as a new clinicopathological entity in an article describing a series of ulcerative lesions with histological features, such as Hodgkin lymphoma associated with several types of immunosuppression (23).

Some authors have suggested that the presence of multiple painful oral ulcers covered by a necrotic pseudomembrane in medically compromised patients may be associated with HSV, CMV or HSV (16). In this review, two patients (8.33%) tested positive for HSV, 1 (4.17%) for CMV, and 5 (20.83%) for EBV.

An elevated level of liver enzymes, usually transaminases, is found in up to 70% of patients in the first 2 to 4 years of treatment. Liver involvement was observed in 3 patients (12.5%), although in 11 patients (45.83%), the state of their liver was not specified. Kidney failure was more common than liver failure; it was recorded in 5 patients (20.83%). Myelosuppression is the most feared adverse effect. Among the most common entities are thrombocytopenia, leukopenia and pancytopenia (8). This result coincides with the results of our review, where 54.17% of patients presented some form of myelosuppression.

Some authors have reported ulcers induced by MTX in patients with preexisting folate deficiency. The elevated levels of MTX in saliva acting topically are considered the triggers of developing lesions. The measurement of MTX excreted in saliva has been proposed as a useful predictor of oral ulceration (8).

Most authors agree that the healing time of the ulcers is complete three weeks after stopping the medication (19). In our study, the mean healing time was 19.9 days. This average healing time cannot be taken as a meta-analysis calculation since all the included studies are related to a report case or series of cases, and the heterogeneity among the studies is so great that it would not be appropriate to do a meta-analysis with the data provided. The objective of our work has been to carry out a systematic review on the management carried out in patients with oral ulcers by methotrexate at low doses, not a meta-analysis on the healing time or other variables described in this study.

Despite the rapid resolution, we should not forget the seriousness of not taking into account acute toxicity following a painful, non-traumatic oral ulcer that does not cease after 15 days of evolution. In the study by Yélamos *et al.*, of the 28 patients who were taking MTX at low doses and who suffered acute toxicity, 7 died (25%) (9). Another reason for worsening is that oral lesions are painful, which often prevents patients from eating, impairing their general health and decreasing folic acid due to a lack of nutrients (21).

Some studies have shown that folic acid supplementation in patients taking MTX reduces adverse mucosal and gastrointestinal effects by 39% (24,25). Of the patients analysed in this review, only 5 took folic acid at the time the lesion appeared (20.83%), while 14 specified that they did not take it (58.33%).

Pearce and Wilson reviewed a total of 47 cases between 1951 and 1996 and found the simultaneous use of NSAIDs and advanced age as risk factors. (26). The average age of our patients was 65.45 years (SD=13.20) and drug interaction with ibuprofen occurred only in 2 patients with oral ulcers.

The differential diagnosis of ulcerations of the oral mucosa is extensive and includes recurrent aphthous stomatitis, autoimmune disorders, allergic reactions, viral and bacterial infections, coeliac disease, agranulocytosis, syndromic diseases and adverse drug effects. A meticulous history and examination can elucidate the underlying cause (21).

An important interaction that dentists and maxillofacial specialists should consider is that nitrous oxide increases the antifolate effect of MTX and its use as inhalation sedation should be avoided in these patients (27).

-Limitations:

All studies that fulfilled the inclusion criteria were “in relation to a case” and most included patients who were referred or urgently admitted to a maxillofacial or emergency service in the hospital setting. This means that data on the patient’s history depended, to a large extent, on data provided by the patient and not to previous monitoring. In addition, due to the aforementioned characteristics, it was not possible to perform an exhaustive follow-up to assess the patients after the event.

## Conclusions

Evidence on the management of oral ulcers in the oral cavity produced by MTX is scarce due to the heterogeneity of data and the measures adopted in the different studies selected to manage these lesions. Therefore, it seems that this management is relegated to the perception of the clinician rather than to a specific protocol of action. Studies with a longer follow-up duration and an analysis of oral ulcers are necessary to guide different health professionals in their management.
